# Morphological Variation and Inter-Relationships of Quantitative Traits in Enset (*Ensete ventricosum* (welw.) Cheesman) Germplasm from South and South-Western Ethiopia

**DOI:** 10.3390/plants6040056

**Published:** 2017-12-06

**Authors:** Zerihun Yemataw, Alemayehu Chala, Daniel Ambachew, David J. Studholme, Murray R. Grant, Kassahun Tesfaye

**Affiliations:** 1 Southern Agricultural Research Institute, Areka Agricultural Research Centre, P.O. Box 79, Areka 1000, Ethiopia; 2 Department of Microbial, Cellular and Molecular Biology, Addis Ababa University, Addis Ababa 1000, Ethiopia; 3 College of Agriculture, Hawassa University, P.O. Box 05, Hawassa 1000, Ethiopia; alemayehuchala@yahoo.com; 4 Southern Agricultural Research Institute, P.O. Box 06, Hawassa 1000, Ethiopia; ddemissi@my.tnstate.edu; 5 Department of Agricultural and Environmental Sciences, Tennessee State University, Nashville, TN 37209, USA; 6 College of Life and Environmental Sciences, University of Exeter, Geoffrey Pope Building Stocker Road, Exeter EX 4QD, UK; D.J.Studholme@exeter.ac.uk (D.J.S.); M.Grant@warwick.ac.uk (M.R.G.); 7 School of Life Sciences, University of Warwick, Coventry CV4 7AL, UK; 8 Institute of Biotechnology, Addis Ababa University, P.O. Box 1176, Addis Ababa 1000, Ethiopia; kassahun.tesfaye@aau.edu.et; 9 Ethiopian Biotechnology Institute, Ministry of Science and Technology, P.O. Box 32853, Addis Ababa 1000, Ethiopia

**Keywords:** landrace, phenotypic variation, *in situ* and *ex situ* genetic resource conservation

## Abstract

Enset (*Ensete ventricosum* (Welw.) Cheesman) is Ethiopia’s most important root crop. A total of 387 accessions collected from nine different regions of Ethiopia were evaluated for 15 quantitative traits at Areka Agricultural Research Centre to determine the extent and pattern of distribution of morphological variation. The variations among the accessions and regions were significant (*p* ≤ 0.01) for all the 15 traits studied. Mean for plant height, central shoot weight before grating, and fermented squeezed *kocho* yield per hectare per year showed regional variation along an altitude gradient and across cultural differences related to the origin of the collection. Furthermore, there were significant correlations among most of the characters. This included the correlation among agronomic characteristics of primary interest in enset breeding such as plant height, pseudostem height, and fermented squeezed *kocho* yield per hectare per year. Altitude of the collection sites also significantly impacted the various characteristics studied. These results reveal the existence of significant phenotypic variations among the 387 accessions as a whole. Regional differentiations were also evident among the accessions. The implication of the current results for plant breeding, germplasm collection, and *in situ* and *ex situ* genetic resource conservation are discussed.

## 1. Introduction

In Ethiopia, the enset-based farming system is a major agricultural system that serves at least one-fifth of country’s population. The total area covered with enset crop has more than quadrupled over the past 50 years from ~65,000 ha in the 1960s [1] to ~300,000 ha in 2010 [2]. The southern and Oromia regions produce 80% of the crop. The productivity of the crop is very high compared to other root and tuber crops and has the advantage of year round food production, but varies depending on edaphic factors, altitude, cultural practices, and varietal differences [3]. Sustained extensive cultivation of enset in Ethiopia reflects the values attributed to every part of the plant, be it for food, fodder, construction, or ornamental purposes [4,5,6].

Enset (*Ensete ventricosum* (Welw.) Cheesman) is the only species of the genus *Ensete* that is cultivated and consumed as a crop [7]. It belongs to the family Musaceae and is a giant herbaceous monocotyledonous plant consisting of an adventitious root system and underground stem structure known as corm, a pseudostem which is formed from leaf sheaths that extend from the base of the plant, leaves, and inflorescence [8]. Ethiopia is both the center of origin and center of diversity for enset [8]. Enset cultivation has been largely confined to Ethiopia, and genetic improvement of this crop is entirely dependent upon characterization and exploitation of Ethiopian germplasm resources. The main food product, known locally as *kocho*, is obtained by fermenting a mixture of the scraped pulp from the pseudostem, pulverized corm, and a stalk of the inflorescence. The corm can be harvested at almost any stage of the crop and cooked and consumed in the same way as other root and tuber crops, relieving hunger during periods of critical food shortages. *Kocho* can be stored for a long time without spoiling [7].

Given the wide geographical range of its cultivation and regional cultural influences, enset domestication has most likely led to extensive genetic variation in landraces. Taboge [9] found large variability in most of the characteristics assessed on 79 enset accessions collected from various parts of the country, and Bekele et al. [10] distinguished and described 120 distinct enset cultivars that fall into 11 broad clusters differing in maturity time, plant height, pseudostem height, pseudostem circumference, leaf number, leaf sheath number, grated corm, and *bulla* and fibre yield. Similarly, other studies [11,12,13] showed substantial phenotypic variation in enset germplasm for phenologic, morphologic, and agronomic traits. To date, six improved enset varieties have been released from direct selection from farmers’ varieties [14].

**Figure 1:**
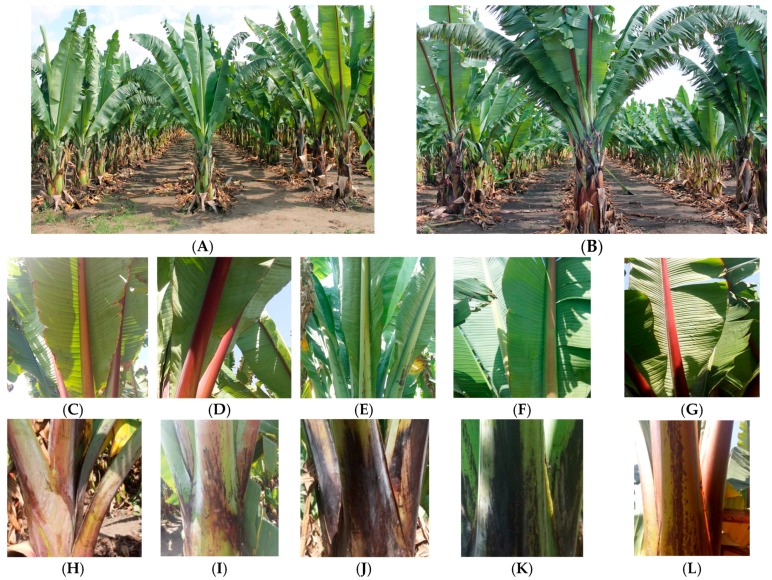
Agro-morphological characterization activity at Areka Agricultural Research Center. (**A**,**B**) Typical arrangement of landraces for phenotyping; (**C**–**G**) Examples of morphological variation in midrib color; (**H**–**L**) Examples of morphological variation in petiole color.

The genetic diversity, which has been collected from different parts of the country, has been conserved *ex situ* in the gene bank of Areka Agricultural Research Centre [14]. However, the value of the conserved germplasm in the bank depends on the information generated through characterization and evaluation for different traits (Figure 1) [15]. Several authors have emphasized that it is important that gene banks have detailed descriptors of morphological characterization and the extent of genetic diversity to provide an essential foundation to explore the genetic variability in breeding programs [16,17]. Frankel and colleagues [18] also stated that genetic resource activities have three phases *viz.* first emphasizing biogeography, taxonomy, and evolution; second conservation; and the third, utilization of germplasm. However, none of these phases has been accomplished satisfactorily in enset. 

To estimate and preserve the genetic variability of enset in Ethiopia, a gene bank was established in 1986 at Areka Agricultural Research Centre, Ethiopia. Attempts have been made to collect and preserve all the possible enset germplasm in Ethiopia and the collection currently comprises 623 enset landraces from 12 major enset growing areas of Ethiopia. A key objective of the National Enset Research Project is to better understand and effectively utilize enset germplasm. The present study has undertaken detailed morphological characterization of part of the collection (based on the availability of planting material), with the objective of accessing the extent and pattern of distribution of morphological variation for quantitative characteristics among a large number of enset accessions.

## 2. Results and Discussion

### 2.1. Univariate Statistics

The analysis of variances indicated significant difference (*p* ≤ 0.01) among accessions, controls (standard checks), and accessions *versus* controls for 13 of the 15 quantitative characteristics assessed. Maturity time, plant height, pseudostem circumference, corm weight before grating, leaf sheath number in accessions (tests) *versus* controls, and pseudostem circumference in controls were not significant (Table 1). These data indicate a high level of morphological variation in Ethiopian enset landraces within each region that could be exploited through breeding programmes (Figure 1). 

For example, the variation for maturity time offers greater flexibility for developing improved varieties suitable for various agro-ecologies of the regions that differ in growing-period length. Similarly, there is potential to develop an early maturing variety by improving traits that correlate to days to maturity. This study detected high levels of variation among accessions in regions of origin based on quantitative characters. The detected morphological variation in enset landraces is strongly influenced by environmental factors. Although morphological diversity in enset has been previously reported [9,11,13], by assessing 15 traits *via* a statistically robust experimental design, this study is the most comprehensive and detailed to date.

Analysis of variance revealed that there were significant differences (*p* ≤ 0.01) among the nine regions of the 387 enset accessions for all characteristics studied (Table 2). The results suggested the occurrence of significant regional differentiation and existence of significant phenotypic variation among the accessions as a whole. Region-wise separation of the variance indicated additional, significant within-region differences (*p* ≤ 0.01) among the populations within Kembata and Hadiya, Gamo Gofa, Wolaita, Sidama, Kaffa, Gurage, Yem special woreda, and west and south-west Shewa for all the 15 characters, and for 14 characteristics within Dawro (Table 2). 

Characteristics which are important for farmers and are used as a selection criterion showed relatively high phenotypic variance. For instance, the within region variation in leaf sheath number, central shoot weight before grating, leaf sheath weight before decortication, fermented unsqueezed *kocho* yield per hectare per year, and fermented squeezed *kocho* yield per hectare per year were all greater than that of plant height, pseudostem height, and pseudostem circumference for all the regions. 

Assuming a significant portion of the underlying phenotypic variation has a genetic basis, it would be possible from a breeding perspective to select for any of the first group of characteristics within a particular region. It was understandable that between regions variance was greater than among accessions pooled over regions and the latter was greater than that among accessions in some regions. Since significant variation was found between regions and among accessions within regions, it would be necessary to collect material from as many regions as possible and to adequately sample the potential local population variation within regions.

The Duncan’s multiple range testing for regional means over all the characteristics is shown in (Table 3). Notably, much more regional differentiation was observed for plant height, central shoot weight before grating, unfermented *kocho* yield per hectare, and fermented squeezed *kocho* yield per hectare per year. More diverse zones favored the development of different quantitative traits. Accessions from Kembata and Hadiya, and Sidama zones showed superior plant height and flowered significantly earlier than those from other regions. The means for number of days to flowering for the accessions from the west and south-west Shewa were significantly higher than those from the other regions (*p* ≤ 0.5). The highest mean number of leaves per plant was also observed for the west and south-west Shewa accessions. This can be explained by the longer induction of floral primordia and more enset leaf formation [9].

Accessions from Dawro, Gamogofa, and Wolaita are inferior in *kocho* yield. Accessions from Sidama are characterized by vigorous plants, being superior in plant height, pseudostem height, pseudostem circumference, leaf length and leaf width. It is a common scenario to observe Sidama farmers feeding their animals with enset leaves. The mean leaf sheath number and leaf sheath weight before decortication (a trait directly related to yield) were high for accessions from Gurage, west and south-west Shewa, and Yem special woreda, though this was not statistically significant compared with those of most of the rest of the regions. 

The accessions from Kembata and Hadiya, and Sidama zones were not significantly different from each other for all the characteristics studied. Thus, the accessions from Kembata and Hadiya, and Sidama could be a good source of early flowering and plant height genes for which there is an urgent need in Ethiopia. Early flowering traits are particularly important for enset production in lowland areas where there is a limited amount of rainfall and a short growing season. 

In general, accessions from Yem special woreda and west and south-west Shewa were characterized by tall pseudostem height and late maturity time. On the other hand, accessions from the other regions were characterized by short pseudostem height and early maturity time, suggesting the possibility of obtaining genes for early flowering and short stature from these landraces. It has been previously speculated that cultural differences have impacted enset selection [4,7,19] and we suggest that this is reflected in these regional phenotypic differences. 

### 2.2. Range and Coefficient of Variance 

The minimum and maximum values of the accession means demonstrated a wide variation among the regions and the accessions within the regions for the characteristics studied (Table 4). Of note, there were large differences between the genotypes in years to flowering. Among the accessions studied, *Azenora* was found to be the earliest (2.1 years) while *Hasa-badadea* was the late maturing (6.3 years) (Supplemental Material). Traits varied from 3- to more than 20-fold. For example, plant height varied more than 3-fold, from 2.14 to 7.71 m; leaves per plant ranged from 5 to 17; leaf sheath number ranged from 11 to 48; leaf sheath weight before decortication ranged from 10 to 200 t ha^−1^ year^−1^; leaf sheath weight after decortication ranged from 3.0 to 85.0 t ha^−1^ year^−1^; fermented unsqueezed *kocho* yield per plant ranged from 2.56 to 42.3 t ha^−1^ year^−1^; fermented squeezed *kocho* yield per plant ranged from 1.26 to 25.14 t ha^−1^ year^−1^. Differences between maximum and minimum values for other characteristics were also large. The wide range in each of the trials studied offers broad opportunities for selecting parents of interest in breeding programs to develop varieties suitable for different agro-ecologies of the country and for different purposes. The broad range noted in phenology as illustrated by maturity time (2.1 to 6.37 year), for example, offers great flexibility for developing varieties suitable for different agro-ecological zones of the country that greatly differ in the length of growing period and/or for use in various cropping systems. Likewise, the variation in plant height, pseudostem circumference, and number of leaf sheaths per plant (Table 4) indicates promising prospects for increasing *kocho* yield in enset. These results support previous studies [9,11] that Ethiopia, with its unique geographic and climatic features, possesses a tremendously high degree of morphological variation for enset.

In the present study, high coefficients of variation were observed between regions and within each region for central shoot weight before grating, leaf sheath weight after decortication, and corm weight before grating (Table 5). 

Notably, accessions from Gurage, Gamo Gofa, Sidama, and Yem special woreda were relatively more variable, demonstrating the tremendous trait variability of within regional enset accessions. Interestingly, the accessions from Kembata and Hadiya, Kaffa, and Dawro had relatively low coefficients of variation for many characteristics, indicating relatively high within region uniformity. The different levels of regional variability of a particular characteristic could be due to differences in natural adaptive selection, a specific selective force, or reflect the impact of human selection. Similar results were reported in tetraploid and hexaploid wheat [20] and in tetraploid wheat [21].

Analysis of the diversity pattern among the enset accessions revealed considerable morphological variations between and within regions. Our results also provided scientific evidence for the occurrence of significant geographical variation and corroborated the idea that regions have a high variation for enset in Ethiopia. The overall patterns of similarity or difference between regions seemed to depend on environmental factors such as rainfall, temperature, length of growing season, and altitude. Similar results have been reported in barley [22,23] and tef [24,25].

### 2.3. Bivariate Statistics

Breeders aim to select superior genotypes on the basis of phenotypic expression. However, for the quantitative characters, genotypes are influenced by environment, thereby affecting the phenotypic expression. Information regarding the nature and extent of association of morphological characteristics to accessions would be helpful in selecting desirable traits and improving yield, a complex characteristic for which direct selection is not effective.

Phenotypic correlation coefficients for the 15 quantitative characteristics were computed for all data (Table 6), for between regions (Table 7) and for within regions (data not shown). The matrix developed for correlation coefficients for all the data showed a significant positive correlation of fermented squeezed *kocho* yield per hectare per year with twelve other characteristics and negative correlation with maturity time. Days to maturity had a positive phenotypic correlation with pseudostem height (0.15), pseudostem circumference (0.12), and corm weight before grating (0.39) (Table 6). This is in agreement with a previous report that *kocho* yield was positively and significantly correlated with plant height, pseudostem circumference, leaf sheath number, and leaf sheath weight [9]. Characteristics that are positively correlated phenotypically are useful in conventional breeding techniques because selection or breeding for one characteristic will likely improve or influence the others. 

Eleven of the 15 characteristics also showed positive correlations with altitude of the collection sites (Table 7). Altitude had a positive and significant correlation with maturity time, pseudostem height, pseudostem circumference, and leaf height (Table 7). As previously discussed [26], ecological characteristics have influenced the genotypic constitution of landraces during domestication, and hence a relationship exists between the agro-ecology at the collection site and the morphological characteristics of the landraces. Thus, positive correlation between collection site variables and plant characteristics would suggest that the variation between accessions is related to agro-ecological variations among the collection sites [27]. The correlation coefficients between leaf number, unfermented *kocho* yield per hectare per year, fermented squeezed *kocho* yield per hectare per year, and altitude were negative but non-significant, indicating that other environmental factors (other than altitude) and/or non-environmental factors might account for the variation for these particular characters.

Knowledge of the magnitudes and the direction of the correlation coefficient between quantitative characteristics would assist the interpretation of the patterns of variation. Within the limits of experimental error and environmental effects, high correlation coefficients among characteristics may reflect a common underlying element of genetic control, or else the impact of unlinked genetic characteristics responding similarly to geographic variation in selection pressures [20,28]. The between-region (also called inter-region) correlation coefficient among the characteristics measures the concordance of their patterns of regional variation, while the within-region (also called intra-region) correlation coefficient measures the association arising from genetic factors not affected by regional variation [28]. 

Since this study showed significant positive correlations intra-regionally for some characteristic combinations, it would imply correlations among the various characteristics had a genetic basis. However, it appeared that similar response to regional variation was playing a greater role than common genetic control as shown by the much more significant and moderate to high correlation coefficients inter-regionally than intra-regionally. 

Correlations among characteristics can help plant breeders identify easily measured characteristics that could be used as indicators of more important (but more complex-to-score) traits. They are also useful in pointing out the possibility and limitation of simultaneous selection of desirable characteristics [29].

The large phenotypic variation observed in this study and previous studies [11] in enset germplasm could be ascribed to many factors. One important factor is the fact that enset is grown in many different environmental conditions, being influenced by rainfall, temperature, altitude, growing period, and edaphic factors. Other factors such as linguistic, cultural, historical, and economic system differences among the people who are cultivating enset [4,6,30,31] likely contribute to its variation. The various physical, biological, and human factors , as well as complex interactions among such factors all seem to have contributed to the wide range of variation of the current enset accessions in the country. 

## 3. Materials and Methods

### 3.1. Description of the Study Site

Enset accessions were evaluated at the Areka Agricultural Research Center, Ethiopia which hosts the coordination of the National Enset Improvement Program and is situated in the heart of one of the major enset producing areas of the country. The Center is located at 7°09′ N latitude and 37°47′ E longitude at an elevation range of 1750 to 1800 m above sea level (m a.s.l.). The soil is silt loam with pH of 4.8 to 5.6 and low to medium organic matter content (2.65–5.67%). The total amount of rainfall for the study period (2012–2017) was 1539 mm, and minimum and maximum mean temperatures were 14.5 °C and 25.8 °C, respectively. Thus, the weather conditions were within the normal range for the growth and development of enset crop in the test area. 

### 3.2. Plant Materials and Study Design

The plant materials used for this study consisted of 387 accessions obtained from a single mother corm, of which 381 were enset landraces derived from nine different regions (Table 8) and six standard controls (released varieties) (Yanbule, Gewada, Endale, Zereta, Kellisa and Messena) [14] conserved ex situ by the Southern Agricultural Research Institute of Agricultural Research at Areka. 

Enset predominantly grows between an altitudinal range of 1200–3100 m a.s.l. It grows particularly well at elevations between 2000 and 2750 m a.s.l. [32], and occasionally plants can also be found at lower altitudes [33]. Based on the altitude at the collection site, the 387 accessions were divided into four sets. These were (i) 44 lowland accessions (<2000 m a.s.l.); (ii) 138 intermediate accessions (2001–2400 m a.s.l.); (iii) 178 highland accessions (2401–2800 m a.s.l.); and (iv) 27 extreme highland accessions (>2800 m a.s.l.). Mean data of the landraces including the regions and altitudes of collection and the vernacular names are summarized in Supplemental Material. 

The experiment was carried out in an augmented design [34] consisting of three blocks in which the 381 landraces were planted in un-replicated plots, and the six standard checks were replicated three times (once in each block) to estimate error variance. Eight suckers of each of the 387 accessions were planted in two rows with intra and inter-row spacing of 3 m and 1.5 m, respectively. All pre- and post-stand establishment management practices were performed as per [11,35]. 

### 3.3. Quantitative Traits and Data Recording

Data were collected for a total of 15 important quantitative (metric or count) pheno-morphological, and agronomic traits (Table 9). All the traits were measured based on published procedures [9,11] from the middle four plants of each accession.

### 3.4. Statistical Analysis

We evaluated the germplasm using an Augmented Incomplete Block design with six standard check varieties *viz*. *Yanbule*, *Gewada*, *Zereta*, *Kellisa*, *Endale,* and *Messena* in three blocks. For all traits assessed on individual plants, the means of the four sample plants from each row were used for analyses. All statistical analyses were done using SAS software [36]. Data were analyzed using the restricted maximum likelihood (REML) model to fit a mixed model with standard controls and experimental site as a fixed effect and non-replicated accessions as random effects [37]. The standard controls were used for estimating error variance in mixed models. The REML model produced best linear unbiased predictions (BLUPs), which can handle unbalanced data while accounting for differences in the amount of data available for each accession [38]. A mixed model procedure was employed to fit analysis of variance of the form:

Y_ij_ = µ + L_i_ + β_j_ + e_ij_

where: Y_ijk_ is the response variable, µ is the general mean, L_i_ is the fixed effect of *i*th standard checks, and random effect of accessions, β_j_, is the random effect of the *i*th block, and e_ij_ is the random errors.

Analysis of variance for the region was made for 15 quantitative characteristics as described by [21]. The mean squares of the nine regions were tested against pooled mean squares of accessions within regions. The pooled mean squares for accessions within regions of origin and the mean squares of accessions within each region were tested against the pooled within region error mean squares.

Means, ranges of means, and percent coefficient of variation (computed as a ratio of standard deviation of each characteristic to the corresponding entire data mean and expressed as a percentage) for all the characteristics were computed for each region of origin and for all the data. The regional means were compared using Duncan’s multiple range testing [39]. 

Pearson correlation coefficients among the characteristics were computed at three levels following published procedures [20,28,39]. First correlations of all the characteristics were assessed based on the mean of the 387 enset accessions, then, the correlation among regions was computed using the means of characteristics for each region. Lastly, a series of intra-region correlation coefficients were obtained for each region using the accession means from that region for the characters.

## 4. Conclusions

Overall, this detailed phenotypic study provided confirmation of widespread statistically significant phenotypic variation in traits at both regional and within-region levels. We predict that the diverse agro-ecology of Ethiopia coupled with the long years of cultivation of the crop under a variety of socio-economic and cultural situations are a major factor in the evolution of the highly diverse phenotypes observed. These findings generally indicate that future enset germplasm collection and conservation strategies cannot easily discriminate among the different regions and altitude zones. With regard to utilization of our diverse germplasm of enset, the study as a whole confirmed the presence of considerable quantitative trait diversity, which can be exploited in the genetic improvement of the crop. The enormous variation identified will provide breeders with new opportunities for breeding and selection of improved enset genotypes.

## Figures and Tables

**Table 1:** Analysis of variance of 15 quantitative traits.

**Source**	**DF**	**MT**	**PLHT**	**PSHT**	**PSCIR**	**LFNO**	**LFL**	**LFWTH**	**LFSTHNO**	**LFSTHBD**
Block	2	2.84 ***	1.32 ***	0.05 *^NS^*	0.09 *^NS^*	8.01 ***	16.73 ***	0.29 ***	32.7 ***	11,484 ***
Accessions	386	4.33 ***	3.92 ***	0.51 ***	0.92 ***	14.08 ***	1.42 ***	0.04 ***	57.8 ***	3789 ***
Test (Landraces)	380	4.29 ***	3.92 ***	0.51 ***	0.93 ***	14.02 ***	1.42 ***	0.04 ***	58.3 ***	3809 ***
Control (Standard Checks)	5	7.95 ***	4.43 ***	0.59 ***	0.23 *^NS^*	19.87 ***	1.81 ***	0.02 ***	36.6 ***	2478 ***
Tests vs. Controls	1	0.05 *^NS^*	0.15 *^NS^*	0.18 ***	0.002 *^NS^*	13.02 ***	6.59 ***	0.21 ***	0.68 *^NS^*	7515 ***
Error	1207	0.06	0.36	0.19	0.83	1.09	0.29	0.08	1.88	12.74
CV		5.67	6.75	11.18	70.1	10.55	8.48	12.2	9.68	19.83
**Source**	**DF**	**LFSTHAD**		**CSBG**	**CORMBG**		**UFK**	**UNSQKOCH**		**SQKOCHO**
Block	2	2832.7 ***		74.87 ***	88.09 ***		119.6 *	185.00 ***		70.4 ***
Accessions	386	882.68 ***		160.9 ***	682.3 ***		1206 ***	186.30 ***		90.9 ***
Test (Landraces)	380	888.01 ***		162.4 ***	672 ***		1213 ***	187.60 ***		91.2 ***
Control (Standard Checks)	5	545.37 ***		57.97 ***	1597 ***		904.9 ***	118.60 ***		86.6 ***
Tests vs. Controls	1	1334.5 ***		161.2 ***	28.62 *^NS^*		342.6 ***	168.60 ***		86.0 ***
Error	1207	5.94		2.95	4.52		5.19	2.37		1.85
CV		19.02		17.98	16.12		14.18	15.64		17.6

MT = Maturity time, PLHT = Plant height (m), PSHT = Pseudostem height (m), PSCIR = Pseudostem circumference (m), LFNO = Leaf number, LFL = Leaf length (m), LFWTH = Leaf width (m), LFSTHNO = Leaf sheath number, LFSTHBD = Leaf sheath weight before decortication (kg), LFSTHAD = Leaf sheath weight after decortication (kg), CSBG = Central shoot weight before grating (kg), CORMBG = Corm weight before grating (kg), UFK = Unfermented *kocho* yield per hectare per year, UNSQKOCH = Fermented unsqueezed *kocho* yield per hectare per year, SQKOCHO Fermented squeezed *kocho* yield per hectare per year; *^NS^*: not significant; *: significant at *p* ≤ 0.5 (5%), ***: and significant at *p* ≤ 0.01 (0.1%).

**Table 2:** Analysis of variance for 9 regions based on 15 quantitative characteristics in enset germplasm.

**Source**	**DF**	**MT ^a^**	**PLHT**	**PSHT**	**PSCIR**	**LFNO**	**LFL**	**LFWTH**	**LFSTHNO**
Region	8	16.38 ***	12.95 ***	0.34 ***	0.13 ***	8.79 ***	1.07 ***	0.02 ***	49.4 ***
Pooled Accessions within Region	378	0.84 ***	0.75 ***	0.12 ***	0.05 ***	3.45 ***	0.3 ***	0.01 ***	14.01 ***
**Accessions within**									
Kembata and Hadiya	77	4.09 ***	2.96 ***	0.49 ***	0.11 ***	15.49 ***	1.19 ***	0.03 ***	31.73 ***
Dawro	54	4.88 ***	4.29 ***	0.62 ***	0.24 ***	13.61 ***	1.81 ***	0.05 ***	118.86 ***
Gamogoffa	44	3.73 ***	3.51 ***	0.67 ***	0.32 ***	9.87 ***	1.15 ***	0.03 ***	29.04 ***
Wolaita	35	4.28 ***	3.50 ***	0.27 ***	0.17 ***	17.54 ***	1.35 ***	0.03 ***	42.30 ***
Sidama	40	3.44 ***	2.14 ***	0.22 ***	0.19 ***	13.86 ***	0.86 ***	0.04 ***	31.36 ***
Gurage	36	2.53 ***	2.50 ***	0.26 ***	0.13 ***	8.75 ***	0.90 ***	0.02 ***	137.33 ***
Yem special woreda	39	1.61 ***	3.44 ***	0.45 ***	0.15 ***	8.93 ***	0.94 ***	0.01 ***	45.69 ***
West and SW Shewa	31	1.44 ***	1.03 ***	0.79 ***	0.14 ***	18.40 ***	1.31 ***	0.02 ***	41.24 ***
Kaffa	22	1.86 ***	2.48 ***	0.30 ***	0.38 ***	20.47 ***	0.98 ***	0.05 ***	17.50 ***
**Source**	**DF**	**LFSTHBD ^a^**	**LFSTHAD**	**CSBG**	**CORMBG**	**UFK**	**UNSQKOCH**		**SQKOCHO**
Region	8	2832.22 ***	732.93 ***	170.84 ***	255.97 ***	2831.53 ***	233.82 ***		125.33 ***
Pooled Accessions within Region	378	741.37 ***	176.87 ***	37.68 ***	162.74 ***	223.7 ***	38.61 ***		19.38 ***
**Accessions within**									
Kembata and Hadiya	77	3457.22 ***	827.10 ***	103.72 ***	675.21 ***	1289.52 ***	195.17 ***		83.93 ***
Dawro	54	3262.67 ***	1030.80 ***	158.48 ***	657.74 ***	1016.38 ***	133.44 *^NS^*		96.93 ***
Gamogoffa	44	2549.44 ***	552.37 ***	290.40 ***	839.27 ***	732.30 ***	126.14 ***		63.82 ***
Wolaita	35	2307.94 ***	451.31 ***	102.54 ***	377.69 ***	971.23 ***	190.72 ***		79.39 ***
Sidama	40	3020.00 ***	636.33 ***	111.05 ***	811.09 ***	1271.21 ***	295.42 ***		150.18 ***
Gurage	36	2400.18 ***	794.97 ***	89.20 ***	607.69 ***	1207.37 ***	200.91 ***		100.59 ***
Yem special woreda	39	3239.8 ***	698.68 ***	146.53 ***	355.21 ***	360.11 ***	34.45 ***		27.87 ***
West and SW Shewa	31	2211.52 ***	372.71 ***	107.49 ***	413.04 ***	105.55 ***	55.71 ***		36.89 ***
Kaffa	22	3794.08 ***	692.86 ***	335.26 ***	895.62 ***	256.46 ***	79.36 ***		45.43 ***

^a^ See the materials and methods section for the abbreviations of the characters; *^NS^:* not significant; ***: and significant at *p* ≤ 0.01 (0.1%).

**Table 3:** Regional means for 15 characteristics in enset.

**Region**	**MT ^a^**	**PLHT**	**PSHT**	**PSCIR**	**LFNO**	**LFL**	**LFWTH**	**LFSTHNO**	
Kembata and Hadiya	3.98d	5.71ab	1.76b	1.16b	10.36b	3.49ab	0.65ab	19.43abc	
Dawro	4.54c	5.32bc	1.68b	1.19ab	9.98b	3.24bc	0.67ab	20.16ab	
Gamogoffa	4.57c	5.31bc	1.72b	1.18ab	10.07b	3.27bc	0.65ab	17.73c	
Wolaita	4.11c	5.13c	1.61b	1.13b	10.34b	3.24c	0.65ab	17.99c	
Sidama	3.82d	5.79a	1.78ab	1.11b	10.08b	3.51ab	0.69a	18.35bc	
Gurage	3.82d	5.45abc	1.64b	1.18ab	10.22b	3.34bc	0.62b	20.24a	
Yem special woreda	5.33ab	5.39abc	1.72b	1.27a	10.51b	3.43ab	0.66ab	20.4a	
West and SW Shewa	5.56a	4.41d	1.94a	1.28a	11.62a	3.68a	0.68a	20.68a	
Kaffa	5.02b	3.77e	1.62b	1.18ab	10.72b	3.12c	0.66ab	18.96abc	
**Region**	**LFSTHBD ^a^**	**LFSTHAD**	**CSBG**	**CORMBG**	**UFK**	**UNSQKOCH**			**SQKOCHO**
Kembata and Hadiya	69.74a	30.06ab	15.41cd	28.91ab	43.18a	16.24a			10.51cde
Dawro	63.36ab	30.97ab	16.23bcd	26.71ab	35.49bc	12.17b			8.94de
Gamogoffa	50.17b	23.78c	14.91cd	27.48ab	33.86c	12.2b			8.32e
Wolaita	51.38b	24.27c	13.7d	24.27b	35.31bc	13.38b			8.79de
Sidama	60.01ab	28.67bc	15.17cd	25.9b	41.28ab	16.57a			10.96bcd
Gurage	62.17ab	29.42abc	15.93bcd	25.02b	45.85a	16.7a			11.68bc
Yem special woreda	71.1a	35.76a	18.83ab	29.63ab	23.07d	12.39b			9.38de
West and SW Shewa	71.85a	34.91ab	20.77a	33.02a	25.47d	17.37a			13.03ab
Kaffa	54.13b	29.71abc	17.27bc	27.91ab	23.81d	18.11a			13.79a

^a^ See the materials and methods section for the abbreviations of the characters; Means of each characteristic followed by the same letter were not significantly different at *p* ≤ 0.05 (5%) according to Duncan’s multiple range test.

**Table 4:** Ranges of accessions means for 15 quantitative characteristics in enset germplasm by region of origin.

**Region**	**MT ^a^**	**PLHT**	**PSHT**	**PSCIR**	**LFNO**	**LFHT**	**LFWTH**	**LFSTHNO**
Kembata and Hadiya	2.07–6.00	3.92–7.61	1.08–2.86	0.75–1.55	5.00–14.50	2.04–4.67	0.42–0.93	13.50–26.50
Dawro	2.5–6.02	2.29–7.35	0.96–3.61	0.54–1.66	4.96–14.50	1.05–4.62	0.33–0.91	11.00–37.00
Gamogoffa	2.3–5.95	2.89–7.15	0.97–2.84	0.78–2.00	6.00–13.20	2.23–4.40	0.43–0.86	12.00–23.00
Wolaita	2.4–5.88	3.32–7.09	1.09–2.11	0.83–1.56	6.50–17.00	1.72–4.32	0.43–0.87	13.00–25.50
Sidama	2.32–5.78	3.68–7.26	1.29–2.16	0.80–1.59	6.25–14.50	2.41–4.51	0.48–0.94	13.00–25.50
Gurage	2.71–5.73	3.01–6.77	1.00–2.09	0.86–1.58	8.00–12.75	2.43–4.24	0.42–0.78	12.50–48.50
Yem special woreda	3.62–6.37	4.15–7.71	1.11–2.46	0.89–186	7.00–13.25	2.43–4.68	0.56–0.82	12.00–27.75
West and SW Shewa	3.06–5.99	3.51–5.30	0.89–3.17	0.84–1.57	8.25–16.25	2.01–4.69	0.52–0.80	12.50–25.50
Kaffa	3.71–5.98	2.14–5.01	1.14–2.33	0.58–1.81	7.50–17.00	2.17–4.20	0.33–0.84	15.70–22.25
**Region**	**LFSTHBD ^a^**	**LFSTHAD**	**CSBG**	**CORMBG**	**UFK**	**UNSQKOCH**		**SQKOCHO**
Kembata and Hadiya	20.46–200.63	11.96–84.88	6.46–29.12	4.96–69.46	11.98–95.47	5.08–40.25		3.20–23.17
Dawro	9.96–137.30	2.96–84.24	1.96–32.38	6.46–62.96	7.06–69.57	1.88–26.98		1.26–20.12
Gamogoffa	13.71–127.21	7.46–66.96	5.96–60.46	8.21–72.46	10.49–83.52	3.28–30.65		1.72–21.27
Wolaita	11.96–110.46	5.46–48.96	3.96–26.96	10.46–46.46	8.40–72.17	2.56–34.08		1.52–21.20
Sidama	25.21–143.96	12.71–63.96	7.96–29.96	7.46–87.96	20.27–82.84	5.82–42.30		2.82–25.14
Gurage	32.96–133.71	10.71–60.96	7.46–28.46	9.46–74.46	17.23–96.96	6.97–33.94		4.25–24.55
Yem special woreda	27.71–151.88	16.21–71.55	8.76–39.71	12.85–65.21	11.65–54.46	7.04–20.35		4.78–16.34
West and SW Shewa	22.51–116.11	13.71–50.06	8.71–32.21	16.14–67.96	16.71–40.92	10.65–27.19		7.90–19.78
Kaffa	20.49–131.96	12.34 64.80	7.55–44.46	13.47–67.29	11.21–47.13	12.42–27.15		9.21–20.15

^a^ See the materials and methods section for the abbreviations of the characters.

**Table 5:** Percent of coefficients of variation for 15 quantitative characteristics in enset germplasm by region of origin.

Region	MT ^a^	PLHT	PSHT	PSCIR	LFNO	LFHT	LFWTH	LFSTHNO	LFSTHBD	LFSTHAD	CSBG	CORMBG	UFK	UNSQKOCH	SQKOCHO
Kembata and Hadiya	25.34	15	19.67	14.13	18.89	15.51	14.63	14.23	42.29	43.48	32.86	44.63	41.53	42.8	43.36
Dawro	24.61	19.64	23.62	20.8	18.64	20.97	17.34	27.24	45.22	51.87	39.08	48.46	45.14	47.9	55.25
Gamogoffa	21.18	17.65	22.8	24.04	15.6	16.72	12.53	15.19	50.32	49.41	57.16	52.71	39.96	46.03	48.01
Wolaita	25.17	18.23	16.12	18.49	20.24	18.54	14.14	18.06	46.75	43.76	36.97	40.03	44.13	51.62	50.67
Sidama	24.31	12.65	13.07	19.77	18.45	13.22	14.57	15.26	45.79	44.01	34.74	54.97	43.18	51.87	52.03
Gurage	20.84	14.55	15.44	15.16	14.46	14.35	10.77	28.94	39.4	47.91	29.65	49.27	37.89	42.42	42.93
Yem special woreda	11.92	17.2	19.47	15.96	14.22	13.78	8.68	16.57	40.02	36.96	32.15	39.76	41.13	23.67	28.16
West and SW Shewa	10.81	11.51	22.98	14.43	18.45	15.5	9.43	15.53	32.72	27.65	24.95	30.77	20.17	21.5	23.31
Kaffa	13.62	20.84	16.78	26.22	21.1	15.93	17.48	11.03	6.42	44.29	53.01	53.62	33.62	24.59	24.35

^a^ See the materials and methods section for the abbreviations of the characters.

**Table 6:** Simple correlation coefficients between 15 quantitative characteristics and with altitudes of the collection site based on the mean for 387 enset accessions.

Caption	MT ^a^	PLHT	PSHT	PSCIR	LFNO	LFHT	LFWTH	LFSTHNO	LFSTHBD	LFSTHAD	CSBG	CORMBG	UFK	UNSQKOCH	SQKOCHO	ALT
MT	1															
PLHT	−0.28 **	1														
PSHT	0.15 **	0.41 **	1													
PSCIR	0.18 **	0.38 **	0.32 **	1												
LFNO	−0.15 **	0.16 **	0.20 **	0.36 **	1											
LFHT	0.04	0.30 **	0.37 **	0.33 **	0.24 **	1										
LFWTH	−0.03	0.40 **	0.41 **	0.50 **	0.28 **	0.39 **	1									
LFSTHNO	−0.06	0.20 **	0.16 **	0.42 **	0.46 **	0.18 **	0.25 **	1								
LFSTHBD	0.04	0.47 **	0.44 **	0.69 **	0.38 **	0.37 **	0.52 **	0.54 **	1							
LFSTHAD	0.07	0.44 **	0.41 **	0.65 **	0.35 **	0.35 **	0.52 **	0.45 **	0.88 **	1						
CSBG	0.07	0.26 **	0.37 **	0.60 **	0.38 **	0.28 **	0.47 **	0.35 **	0.66 **	0.68 **	1					
CORMBG	0.39 **	0.36 **	0.42 **	0.58 **	0.08	0.22 **	0.33 **	0.14 **	0.54 **	0.57 **	0.48 **	1				
UFK	−0.52 **	0.56 **	0.24 **	0.40 **	0.30 **	0.26 **	0.41 **	0.34 **	0.57 **	0.57 **	0.43 **	0.29 **	1			
UNSQKOCH	−0.33 **	0.31 **	0.33 **	0.41 **	0.42 **	0.26 **	0.41 **	0.37 **	0.59 **	0.64 **	0.53 **	0.35 **	0.70 **	1		
SQKOCHO	−0.20 **	0.27 **	0.34 **	0.46 **	0.42 **	0.26 **	0.40 **	0.39 **	0.61 **	0.66 **	0.55 **	0.40 **	0.61 **	0.93 **	1	
ALT	0.10 *	0.043	0.10 *	0.12 *	−0.01	0.14 **	0.05	0.07	0.06	0.06	0.04	0.02	−0.07	−0.09	−0.07	1

ALT = Altitude; *: significant at *p* ≤ 0.5 (5%); **: significant at *p* ≤ 0.1 (1%); ^a^ See the materials and methods section for the abbreviations of the characters.

**Table 7:** Inter-region simple correlation coefficients among 15 quantitative characteristics (based on the mean of the 9 regions of origin).

Caption	MT ^a^	PLHT	PSHT	PSCIR	LFNO	LFHT	LFWTH	LFSTHNO	LFSTHBD	LFSTHAD	CSBG	CORMBG	UFK	UNSQKOCH	SQKOCHO
MT	1														
PLHT	−0.65	1													
PSHT	0.37	0.04	1												
PSCIR	−0.14	0.26	0.27	1											
LFNO	0.71 *	−0.65	0.61	0.09	1										
LFHT	0.16	0.27	0.91 **	0.35	0.52	1									
LFWTH	0.39	−0.25	0.52	−0.17	0.31	0.310	1								
LFSTHNO	0.44	−0.11	0.34	0.19	0.46	0.420	−0.03	1							
LFSTHBD	0.33	0.17	0.62	0.50	0.45	0.74 *	0.15	0.85 **	1						
LFSTHAD	0.51	−0.08	0.52	0.43	0.53	0.580	0.25	0.86 **	0.95 **	1					
CSBG	0.85 **	−0.49	0.59	0.01	0.81 **	0.510	0.36	0.76 *	0.69 *	0.79 *	1				
CORMBG	0.79 *	−0.37	0.78 *	0.35	0.79 *	0.620	0.40	0.54	0.66	0.72 *	0.87 **	1			
UFK	−0.93	0.71 *	−0.15	0.26	−0.59	0.050	−0.44	−0.20	−0.09	−0.33	−0.67	−0.59	1		
UNSQKOCH	−0.048	−0.42	0.27	0.18	0.51	0.290	0.15	0.17	0.17	0.25	0.27	0.22	0.08	1	
SQKOCHO	0.27	−0.65	0.26	0.03	0.65	0.210	0.22	0.35	0.24	0.39	0.54	0.39	−0.22	0.93 **	1

*: significant at *p* ≤ 0.5 (5%); **: significant at *p* ≤ 0.1(1%); ^a^ See the materials and methods section for the abbreviations of the characters.

**Table 8:** Regions of origin, altitude, and numbers of landraces used for this study. m a.s.l. = meters above sea level.

Collection Region or Altitude Zone	Total No. Populations
**Region**	
Kembata and Hadiya	78
Dawro	55
Gamogoffa	45
Wolaita	36
Sidama	41
Gurage	37
Yem special woreda	40
West and SW Shewa	32
Kaffa	23
**Altitude Zones**	
≤2000 m a.s.l.	44
2001–2400 m a.s.l.	138
2401–2800 m a.s.l.	178
>2800 m a.s.l.	27

**Table 9:** List of quantitative characteristics recorded in the study.

Quantitative Trait	Code	Description
Maturity time	MT	Number of years from transplanting up to harvesting.
Plant height (m)	PLHT	Measurement from ground level to the tip of the longest leaf at flowering.
Pseudostem height (m)	PSHT	Measurement from ground level to the start of the petioles.
Pseudostem circumference (m)	PSCIR	Measurement at the middle height of the enset pseudostem.
Leaf number	LFNO	The number of 50% green and 50% unrolled leaves.
Leaf length (m)	LFL	Measurement of all functional leaves from the end of the petiole to the tip of the leaf and their mean was taken for analysis.
Leaf width (m)	LFWTH	Measurement of the widest part of all functional leaf blades just below flag leaf and their mean was taken for analysis.
Leaf sheath number	LFSTHNO	Count of all decorticatable leaf sheaths obtained from each plant at harvest.
Leaf sheath weight before decortication (kg)	LFSTHBD	Weight of all leaf sheaths for each plant before decortication and measured before decortication
Leaf sheath weight after decortication (kg)	LFSTHAD	The weight of pulp for each plant after decortication and measured after decortication.
Central shoot weight before grating (kg)	CSBG	The weight of central shoot after the inflorescence removed is measured before grating.
Corm weight before grating (kg)	CORMBG	The weight of corm after fibrous roots removed and measured before grating.
Unfermented *kocho* yield per hectare per year	UFK	*Kocho* yield comprising the mixture of decorticated leaf sheath, grated central inflorescence bearing shoot, and corm immediately after processing.
Fermented unsqueezed *kocho* yield per hectare per year	UNSQKOCH	The unfermented *kocho* yield is left in the pit for some time, usually 30 days, for fermentation. The fermented *kocho* was measured for its weight before squeezing.
Fermented squeezed *kocho* yield per hectare per year	SQKOCHO	The fermented *kocho* yield was squeezed by applying human force to reduce its water as much as possible.

## Supplementary Materials

The following are available online at http://www.mdpi.com/2223-7747/6/4/56/s1. Spread sheet Means of 15 Quantitative traits of the 387 enset accessions tested at Areka.

## Abbreviations

AAU	Addis Ababa University
CSA	Central Statistics Authority
m a.s.l.	Meter above sea level
SARI	Southern Agricultural Research Institute
SNNPRS	Southern Nations, Nationalities and Peoples’ Regional State
